# “Road traffic incidents: an emerging epidemic and can we make an epidemic “U” turn?

**DOI:** 10.1186/1865-1380-8-S1-P10

**Published:** 2015-04-22

**Authors:** Sri Ramula Sai Dilip, Radhika Rani Chandra Taaneru Venugopal

**Affiliations:** 1 Guntur Medical College and Hospital, Guntur, Andhra Pradesh, India

## Objectives

To assess Knowledge, Awareness, practices regarding road safety measures and consequences of Road Traffic Injuries (RTIs) among medical students of Guntur Medical College and Hospital, Guntur.

## Methods

A cross-sectional study was conducted among medical students of Guntur Medical College and Hospital using pretested and semi-structured questionnaire in the month of September 2014. The collected data was entered in MS Excel and analyzed by EPI INFO.

## Results

Among 206 students interviewed, 166 (80.6%) were females and 40 (19.4%) were males. Among the study subjects, 123(59.7%) drive motorcycles but only 83(40.3%) have a license. Majority 121 (58.7%) had witnessed an accident. 201 (97.6%) out of 206 students knew that wearing helmet is safe, but only 89 (43.2%) wear helmets. Only 30.2% of the study group wears a seatbelt. Most of them, 73 (35.4%), have knowledge of traffic symbols.

## Limitations

This study was done on medical students so it cannot be generalized to the public.

## Conclusion

Observations of the study emphasize that there need to enhance knowledge and awareness among medical students through education training to curb the epidemic of RTIs.

